# The Economic Burden of Malaria on Households and the Health System in Enugu State Southeast Nigeria

**DOI:** 10.1371/journal.pone.0078362

**Published:** 2013-11-04

**Authors:** Obinna Onwujekwe, Nkoli Uguru, Enyi Etiaba, Ifeanyi Chikezie, Benjamin Uzochukwu, Alex Adjagba

**Affiliations:** 1 Health Policy Research Group, Department of Pharmacology and Therapeutics, College of Medicine, University of Nigeria Enugu-Campus, Enugu, Nigeria; 2 Department of Health Administration and Management, College of Medicine, University of Nigeria Enugu-Campus, Enugu, Nigeria; 3 Department of Preventive Dentistry, College of Medicine, University of Nigeria Enugu-Campus, Enugu, Nigeria; 4 Department of Community Medicine, College of Medicine, University of Nigeria Enugu-Campus, Enugu, Nigeria; 5 SIVAC (Supporting National Independent Immunization and Vaccine Advisory Committees) Initiative, Agence de Medecine Preventive (AMP), Paris, France; National Institute of Medical Research, United Kingdom

## Abstract

**Background:**

Malaria is the number one public health problem in Nigeria, responsible for about 30% of deaths in under-fives and 25% of deaths in infants and 11% maternal mortality. This study estimated the economic burden of malaria in Nigeria using the cost of illness approach.

**Methods:**

A cross-sectional study was undertaken in two malaria holo-endemic communities in Nigeria, involving both community and hospital based surveys. A random sample of 500 households was interviewed using interviewer administered questionnaire. In addition, 125 exit interviews for inpatient department stays (IPD) and outpatient department visits (OPD) were conducted and these were complemented with data abstraction from 125 patient records.

**Results:**

From the household survey, over half of the households (57.6%) had an episode of malaria within one month to the date of the interview. The average household expenditure per case was 12.57US$ and 23.20US$ for OPD and IPD respectively. Indirect consumer costs of treatment were higher than direct consumer medical costs. From a health system perspective, the recurrent provider costs per case was 30.42 US$ and 48.02 US$ for OPD and IPD while non recurrent provider costs were 133.07US$ and 1857.15US$ for OPD and IPD. The mode of payment was mainly through out-of-pocket spending (OOPS).

**Conclusion:**

Private expenditure on treatment of malaria constitutes a high economic burden to households and to the health system. Removal of user fees and interventions that will decrease the use of OOPS for treatment of malaria will significantly decrease the economic burden of malaria to both households and the health system.

## Introduction

Malaria is a major public health problem in Africa and one of the leading causes of avoidable death, especially in children and pregnant women [1], [2]. It imposes a significant economic burden on Africa which has the greatest number of malaria cases in the world [2], [3]. Africa accounts for 60% of the world's 350–500 million clinical malaria cases and a total malaria cost of US$12 billion according to World Health organization (WHO) estimate in 2000 [2]. Households in Africa spend between $2 and $25 on malaria treatment and between $15 and $20 on prevention each month with consequent loss of resources [3]. The human and economic costs associated with declining quality of life, consultations, treatments, hospitalizations and other events related to malaria are enormous and often lead to low productivity and lost incomes [4]. Malaria is deemed as not only a public health problem but also a deterrent to the socioeconomic growth of a country [5], [6]. The amount spent on malaria in terms of prevention, treatment and loss of productivity can comprise a significant portion of the annual income of poor households [7].

In Nigeria, malaria is holo endemic, with only a small area in the middle belt at a 3% risk of epidemic [8]. Available records show that annually, about 50% of the population suffers from at least one episode of malaria while children under 5 have an average of 2–4 attacks of malaria [9]. It has also been found that over 300,000 die each year of the disease and malaria is responsible for 60% outpatient visits to health facilities, 30% childhood death, 25% of death in children under one year, 11% maternal death [10]. Indeed, malaria constitutes a heavy burden on Nigeria's families, communities, health system, and workforce [11].

Given this magnitude of disease burden, estimating the economic burden of malaria is necessary to provide a basis or platform for advocacy with Ministries of Finance and donors for increased investments in addressing public health problems such as malaria [2]. Data on the economic burden of malaria provide essential information on the magnitude of the problem. They complement disease burden data and can be used to show who bears the economic burden of malaria [7]. Recent studies have shown that although there are equal exposure and incidence of malaria across the socio-economic groups [12], the costs of treating malaria cases vary amongst different socio-economic status (SES) groups and geographical locations and that payments for treating malaria cases were uniformly by out-of-pocket payments [13], [14]. These costs have been shown to be proportionately higher in poor households compared to their income and are catastrophic to poorer households and to rural dwellers [14].

In Nigeria, malaria has been shown to account for over 40% of the total monthly curative healthcare costs incurred by households compared to a combination of other illnesses; the cost of treating malaria and other illnesses depleted 7.03% of the monthly average household income, and treatment of malaria cases alone contributed 2.91% of these costs [15]. Household spending on malaria can be classified into expenditure on prevention and expenditure on treatment. Individual or household direct cost of malaria treatment include direct payment of drugs, consultation, laboratory tests, transportation fees to and from the facility [16] while the indirect cost is the productive time lost due to malaria.

This paper provides new information about the economic burden of malaria treatment borne by households and providers (health system) in Enugu state, southeast Nigeria using a cost of illness approach. It specifically examines the direct medical, direct non-medical and indirect costs per episode of malaria to both the households and the health system.

## Research Methods

### Ethical considerations

Ethical approval for the study was obtained from the Ethics Review Board, University of Nigeria. Each respondent gave a signed informed consent. In addition, the heads of each facility gave an informed consent before data was abstracted from each of them. Consent was obtained from the hospital authorities to use anonymized data extracted from the hospital database for the study. The IRB approved the study based on written consent obtained from the hospital authorities and based on the anonymous nature of the data abstraction. Some patients that were interviewed during the study also had information obtained from their medical records and these respondents gave their consent both for the interviews and the data abstraction. About a quarter of the patients who were interviewed also had data abstracted from their records.

### Study Area

The study was conducted in Enugu State southeast Nigeria. Enugu state has an estimated population of 3.3 million and a total land area of 7,618 sq. km [17]. The state is divided into 17 Local Government Areas (LGAs). The study sites (Achi and Oji urban) are in Oji River LGA. It has seven communities namely Awlaw, Achi-uno, Achi-agu, Oji urban, Ugwuoba, Inyi and Akpugo-Eze [18].

Achi and Oji urban are malaria holo-endemic communities with an average malaria incidence rate of 15% [15]. Achi community has an estimated population of 46,112 people and is divided into 12 villages. Oji urban has a population of 14,026. Enugu state runs a free maternal and child health (FMCH) programme which was started in 2008; children under 5 and pregnant women are offered free medical treatment at the public health facilities in the state. Despite this free treatment, a lot of households still bear a significant cost burden for malaria treatment because of frequent drug stock-outs in public health facilities. There is also the additional non-medical costs (direct and indirect) which is not met by the free programme that are usually borne by households as out of pocket payments.

### Study Design, Sampling, Data collection and Analysis

The study was a cross-sectional study comprising both household and hospital based surveys. The household survey involved households with children less than five years residing in the communities while hospital based survey involved patient exit interviews, retrospective and prospective data abstraction from case records. A pre-tested questionnaire was used to elicit information from patients leaving the health facilities after consultation and treatment for malaria.

A list of all the households with children under five years in the two selected communities was compiled, so as to get a sampling frame. Systematic random sampling was used to select 500 households for the study. The primary caregivers or any other household representatives in each of these households were interviewed.

Six health facilities (1 secondary public hospital, 4 primary healthcare centres and a mission hospital) were purposively selected based on their geographic region and patient load. A total of 125 exit interviews were administered to caregivers of children that had been diagnosed with malaria after consultation and treatment. A proportionate sampling technique was used to assign the number of exit interviews carried out in the respective health facilities. This was complemented with retrospective and prospective data abstraction from 125 in-patient and out-patient case records.

In the household survey, information was collected on the demographic characteristics of the respondents in each selected household, the malaria status of children under the age of five years, cost of treating malaria episode, household assets and monthly food expenditure. Data on transportation costs to and from the facility where treatment was sought and any other costs incurred in the course of seeking treatment for malaria was also collected.

At the facilities, consent was obtained from the hospital authorities to use anonymized data extracted from the hospital database for the study. The IRB approved the study based on written consent obtained from the hospital authorities and based on the anonymous nature of the data abstraction. Some patients that were interviewed during the study also had information obtained from their medical records and these respondents gave their consent both for the interviews and the data abstraction. About a quarter of the patients who were interviewed also had data abstracted from their records.

Similar information as in the household survey was also collected from exit interviews and in addition any costs incurred in treatment of malaria before attending the facility. In-patient data on costs of malaria treatment (drugs, laboratory tests, etc.) were abstracted from case records within a year (11 months retrospective and 1 month prospective). Information on costs to the health care system-drugs, investigations, facility running costs (recurrent and non-recurrent) was abstracted from the accounts records and these were validated by both the head of accounts department and the facility head. The capital cost of the buildings were estimated by an estate valuer and a health economist. Depreciation rates were applied to both buildings and vehicles. Estimation of vehicles and laboratory equipment were not applicable to the health centre facilities as they run only out-patient services.

N.B. Questionnaires will be made available to readers on request.

### Data Analysis

Data analysis was undertaken and frequency distributions analyzed. Summary statistics at both the household and provider levels were computed. For continuous variables, the mean and standard deviation were calculated while numbers and percentage were determined for categorical variables. The direct medical costs that were computed consist of drugs cost, diagnostics, administration fees and other costs incurred as a result of the treatment of malaria. The direct non- medical cost consisted of transport fare only. The household's indirect cost of treatment was the cost attributed to time lost when taking care of a sick child. Principal component analysis (PCA) was conducted to generate a socioeconomic status (SES) index and wealth quintiles based on per capita food expenditure and household asset ownership.

Note: 154.06 Naira = 1USD (CBN, Nigeria exchange rate)

## Results

Table 1 (See Tables section) presents a summary of the socioeconomic and demographic characteristics of the sampled households. Majority of the respondents were females, petty traders and subsistence farmers with a mean age of 33 years. The average household size was 5 people. The number of households with children that had an episode of malaria within the last one month of the date of interview was 288 (57.6%).

**Table 1:** Socio-demographic characteristics of respondents (N = 500).

Variable	Measurement
**Sex**	**n (%)**
Female	484 (96.8)
Male	16 (3.2)
Age (years) : mean (SD)	33 (9.20)
**Main Occupation**	**n (%)**
Petty trading/Artisan	137 (27.4)
Subsistence farmer	109 (21.8)
Large scale private Entrepreneur	48 (9.6)
Self -Employed	39 (7.8)
Government worker-(Public Service)	37 (7.4)
Employed by a private organization	12 (2.4)
Others	80 (16.0)
Unemployed	39 (7.8)

### Household costs of malaria treatment

Table 2 shows that the direct medical cost at 3.05 USD was higher than the direct non- medical cost (0.41 USD). However the indirect cost of treatment was the highest cost (9.11 USD). The average out- patient cost of taking care of a child sick with an episode of malaria was 12.57 USD.

**Table 2:** Out-Patient Department (OPD) Costs.

Variable	Mean(SD)Naira	Mean(SD)US$
**Direct Medical Costs**	469.08 (390.06)	3.05 (2.53)
Drugs	395.28 (362.14)	2.56 (2.35)
Diagnostics	20.42 (97.51)	0.13 (0.63)
Administration fees	46.83 (37.76)	0.30 (0.24)
Others	7.32 (25.57)	0.1 (0.16)
**Direct non medical (Transportation cost)**	64.01 (95.35)	0.41 (0.62)
**Indirect cost (Loss of Income)**	1404.00 (1065)	9.11 (6.91)
**Total-OPD cost per case**	**1937.09**	**12.57**

Table 3 shows that the average cost per case of malaria for In-patient Department (IPD) stay is $23.20 USD. However, the cost attributed to loss of income in taking care of a sick child is the highest contributor (12.88 USD) of total cost followed by direct medical costs (6.73 USD). The patients incurred an average of 3.0 USD seeking treatment elsewhere before being admitted at the health facility, and less than one dollar on transportation costs.

**Table 3:** In Patient Department (IPD) Costs.

Variable	Naira	US$
**Direct medical costs**	1037 (589.02)	6.73 (3.82)
Drugs	766.72 (429.69)	4.97 (2.78)
Diagnostics	187.41 (203.59)	1.21 (1.32)
Administration fees	42.24 (29.56)	0.27 (0.19)
Others	37.93 (80.01)	0.24 (0.5)
**Direct-non medical(Transportation cost)**	92.07 (96.57)	0.59 (0.62)
**Indirect cost (Loss of Income)**	1985.00 (937.77)	12.88 (6.08)
**Costs Before IPD**	420.34 (611.86)	3.0 (3.97)
**Total-IPD cost per case**	**3534.41**	**23.20**

### Provider (Health System) Costs

#### Recurrent costs

The average recurrent provider costs incurred to treat one malaria case in OPD and IPD were $30.42 USD and $48.02 USD respectively (Table 4).

**Table 4:** Cost per case for OPD (n = 125) and IPD (n = 125).

Variable	OPD {Naira (US$)}	IPD {Naira (US$)}
Drugs	286.55 (1.86)	368.20 (2.39)
Medical Supplies	392.85 (2.55)	1095.36 (7.11)
Lab. And Diagnostic tests	215.68 (1.4)	50.83 (0.33)
Health Personnel	3745.97 (24.31)	5663.03 (36.75)
Utilities	46.21 (0.30)	221.85 (1.44)
**Total cost per case**	**4687.26 (30.42)**	**7398.46 (48.02)**

#### Non- recurrent cost (Capital cost)

The capital cost to the provider to treat one case of malaria for a year was $133.07 USD for OPD and $1857.15 USD for IPD (Table 5).

**Table 5:** Annual capital cost for OPD and IPD.

Cost Component	OPD {Naira (US$)}	IPD {Naira (US$)}
Health centre building	20,000 (129.82)	N/A
District Hospital building	N/A	90,000 (584.18)
Furniture	500 (3.25)	1,714.29 (11.13)
Vehicles	N/A	180,000 (1,168.37)
Laboratory Equipment	N/A	14,400 (93.47)
**Total**	**20,500 (133.07)**	**286,114.29 (1857.15)**

N/A: Not Applicable.

#### Cost Summary

The total costs (household, provider-recurrent, provider-capital) for OPD and IPD sum up to $176.04 USD and $1928.37 USD respectively.

## Discussion

This study estimated what an episode of malaria costs for OPD and IPD treatment from the demand and supply side respectively, using the cost of illness approach. The total costs are enormous given the prevalence and the high transmission rate of malaria in this area and also given that a significant proportion of the population live below the World Bank poverty line. With several episodes of malaria, the household and provider recurrent costs are increased further. This high cost burden has also been reported in other studies in Nigeria and some other African countries [2] and also by studies that have used a different approach like the Willingness to Pay approach (WTP) [10], [19]. These total costs get shifted between consumer (households) and provider (health care system) in varying proportions at different times depending on the context.

It was found that indirect costs (i.e. productivity losses for IPD and OPD treatment of malaria) by the respondents were greater than direct medical and direct non medical costs put together for IPD and OPD. This could be partly due to the free maternal and child health care (FMCH) services offered in most public facilities in the state [18] and because care-givers sacrifice their business time, their work hours in the course of taking care of a sick child (person) or in the course of receiving care themselves in the hospital. Hence, in principle and policy, malaria treatment may be free of charge for children and pregnant women but this does not mean that treatment is cost-free from the perspective of the patient and households who may incur costs such as transportation, food expenditure, accommodation and loss of time [20], [21]. In other words, where FMCH is being fully implemented and there is availability of drugs, it only shifts the direct medical cost to the health care system while households are still left with the higher proportion of the cost burden (direct non-medical and indirect costs). These costs may constitute a significant portion of a household's income and may even be catastrophic to poorer households [2], [14], [22].

The treatment cost per IPD case was greater than that for OPD expectedly, as this also includes hospitality costs. However it may have been biased upwards by additional treatment of co- morbidities and other systemic complications that can arise from severe malaria which make patients spend more time in the hospital thereby incurring more costs than out patients [23]. This in turn directly affects the indirect costs of the disease since caregivers will spend more days caring for the sick child causing productivity losses [24]. Costs incurred at other facilities by households before being admitted to the IPD for treatment also contributed to the total costs. These costs are sometimes high because of the treatment seeking pattern in this area where patients tend to attend patent medicine dealers (PMDs) first for malaria treatment, and only go to the hospital when symptoms do not subside [25].

Most of the respondents being farmers and petty traders and most likely poor, may have suffered catastrophic health spending for malaria treatment as a result of these costs borne by households [15] and there may be further skewing against the rural dwellers as has been shown in a different study [14].

The major mode of payment for health services was out of pocket and none of the respondents had health insurance. Although, the national health Insurance scheme was launched in Nigeria years ago to reduce the burden of illness borne by individuals, none of the respondents had any form of health insurance. This goes to emphasize the low financial risk protection in the country and the lack of which will deplete the meagre resources of the poor leaving them impoverished from health spending [12].

Due to the free maternal and child health program (FMCH) going on in the state, the provider bears some recurrent costs for the treatment of malaria. The cost of laboratory and diagnostic tests is higher in OPD than IPD. This is because in OPD most of the tests done are rapid diagnostic tests (RDTs) which the patients do not pay for and so the provider bears the cost while in the IPD, tests carried out are mainly microscopy and this is paid for out of pocket. This means that in areas or states where free MCH is non-functional, additional costs are shifted to the household. This is also sometimes the case in the study areas when the facilities experience drug stock-outs and patients are forced to purchase their drugs from drug shops or other itinerant drug sellers. This further impoverishes poor households.

In health systems where financial risk protection is in operation, the cost of treatment will be predominantly incurred by the provider at the point of need but this is not the case in this study. In urban areas where there has been minimal uptake [26], there may well be some variation in the proportion of who pays for treatment-household or health system.

The cost of malaria treatment is high in the study sites in Nigeria and currently, households bear a greater portion of this cost due to a high level of indirect costs. To some households this may be catastrophic. There is need to buffer this with some sort of financial risk protection mechanisms and the health care system needs to be strengthened to function more effectively and decrease overall consumer and provider economic burden of malaria.

Since free MCH is not operative in all states of Nigeria, these findings may not be generalisable to the whole country. However, given the uniform high disease burden and holo-endemicity, the gross cost burden is likely to be similar. Further studies are required to determine the level to which co-morbidities may have biased the results and also to determine how households cope with these high costs of malaria treatment. Retrospective data abstraction may have been prone to inaccuracy but this was controlled by a one-month prospective in-patient data collection which was comparable.

In conclusion, the cost of treating malaria is high both to the household and to the health system. Where costs are shifted to the health system, they constitute a significant portion of the healthcare budget which could have been efficiently used in other areas of healthcare delivery. Multipronged approaches are needed to address the high level of economic burden of malaria through concerted and sustained malaria preventive efforts; use risk protection mechanisms to buffer both direct and indirect costs of treatment; and health system and policy research for best practices of risk pooling and risk protection mechanisms that are suited to the developing country context.
